# Food security and well-being among older, rural Americans before and during the COVID-19 pandemic

**DOI:** 10.1371/journal.pone.0274020

**Published:** 2022-09-02

**Authors:** Stacey Giroux, Kurt Waldman, Mecca Burris, Julia C. D. Valliant, Angela M. Babb, Philip Stafford, Daniel Fobi, Kamila Czebotar, Daniel C. Knudsen

**Affiliations:** 1 Ostrom Workshop, Indiana University, Bloomington, Indiana, United States of America; 2 Department of Geography, Indiana University, Bloomington, Indiana, United States of America; 3 Department of Anthropology, Indiana University, Bloomington, Indiana, United States of America; 4 CoDesign Commons, Bloomington, Indiana, United States of America; St John’s University, UNITED KINGDOM

## Abstract

The COVID-19 pandemic has impacted many aspects of our lives. Older adults, those with less income or fewer resources, and those living in rural parts of the United States are potentially more vulnerable. To understand the negative impact of COVID-19 on perceived food security, physical and mental health, and loneliness in a sample of older, rural, low-income adults in the United States, we use results from a mailed survey in which residents of four Indiana counties contrasted their status during the early period of the pandemic to their typical pre-pandemic status. We test for significant changes in status and what predicts negative impacts to food security, health, and loneliness. We asked respondents to report on both pre-pandemic and since-pandemic experiences in the instrument, which was administered after the pandemic had begun, in May 2020. We measure food security using the U.S. Household Food Security Survey Module (six-item short form; HFSSM), physical and mental health using the Centers for Disease Control’s Healthy Days Measures (HRQOL-4), and loneliness using the UCLA Revised Loneliness Scale. A binomial test identified significant declines in status for all three measures. Logistic regressions identified factors associated with each of the measures worsening. Fear of going to the store or food pantry was associated with all three measures. Decreased store hours and closed food pantries were associated with lower food security. More education, fewer years of age, being female, decreased income, and stockpiling were associated with more reported days of poor physical or mental health. Fewer years of age, lack of transportation, and eating less often with others were associated with perceived increased loneliness. The pandemic had a negative impact on respondents’ food security, unhealthy days, and loneliness, but different factors were associated with each measure for this population. Our findings provide insight for targeted recovery efforts.

## Introduction

The intersection of age, rurality, and poverty renders certain individuals especially vulnerable to the more insidious effects of COVID-19 [1]. Generally, people with less income living in rural areas have less access to health care, a lower life expectancy at birth, are more likely to be food insecure, suffer poorer health overall, and contend with an increasing mortality penalty [1, 2]. Rural areas are also older and aging faster than urban ones, with close to one-quarter of low-income older Americans living in rural areas [3]. Compared to their urban-dwelling peers, rural older adults are also more likely to face food insecurity, which includes “reduced quality, variety, or desirability of diet” as well as “disrupted eating patterns and reduced food intake” [4], and an increased risk of loneliness, both of which are linked to poorer health outcomes [5, 6].

Having access to enough, healthy food is a minimum requirement for good health. Being unable to reliably procure healthy food, whether due to cost or other issues related to access, can be a source of chronic stress. Such stress, coupled with poorer nutrition, can lead to negative health outcomes. The COVID-19 pandemic fractured many food supply chains and, in conjunction with loss of jobs and income, threatened individuals’ food access and created uncertainty for many about having enough food. At the same time, lockdowns and social distancing meant that people could no longer spend time with others in person, nor could they eat with others in the manner they had previously. Commensality provides regular opportunities for socialization and has also been shown to play a role in good mental health and keeping loneliness at bay [7].

We are beginning to understand some of the effects of COVID-19 and the pandemic more broadly on food security and other health-related measures. Rates of food insecurity in the United States increased [8, 9], although some work has found that older people were less likely to experience pandemic-related food insecurity [10]. Loneliness due to lockdowns increased in general but also specifically among older adults in Indiana [11]. Stress caused by the need for constant risk evaluation, trauma from experiencing the illness and the death of loved ones, and restrictions on social interaction, among other factors, are forecast to impact the mental health of the global population well into the future [12]. Rural areas have seen higher rates of COVID-19 comorbidity, in part because of larger elderly populations who are more susceptible to serious complications arising from infection with COVID-19, but also because of the lack of health care infrastructure and lower rates of health insurance among residents [13]. Overall, rural areas have been projected to be more impacted by the pandemic than major urban areas in the U.S. [14].

In addition to documenting certain effects of the pandemic on a particular population of concern, this paper responds to recent work that recognizes the ways that the pandemic has exposed weaknesses in the nation’s rural health systems, and reiterates the value of place, that is, the attributes of the conditions or contexts in which people are living, in public health [15–17]. Using a cross-sectional, mailed survey instrument, we investigate whether a sample of older adults in rural Indiana viewed three specific aspects of well-being to have been negatively impacted by the COVID-19 pandemic, and if so, what pandemic-related factors predict that negative impact. We use the U.S. Household Food Security Survey Module (HFSSM) short form (six item) [18], two items from the Centers for Disease Control’s Health-Related Quality of Life core module (HRQOL-4) [19], and the shortened version of the Revised UCLA Loneliness Scale, the Three-Item Loneliness Scale [20] to assess food security, physical and mental health (unhealthy days), and subjective isolation, or loneliness, before and during the COVID-19 pandemic. We also developed a set of questions to assess structural and psychological factors that may contribute to changes in these measures since the pandemic began.

We found that overall, scores for food security decreased, and respondents reported having more unhealthy days and feeling more lonely since the pandemic began. Further, different factors were associated with each of the three measures of well-being for this sample, illustrating how complex it will be to continue to address recovery needs in the wake of COVID-19. Priorities will vary demographically and geographically [21], and our results will help practitioners and policymakers plan for the needs of older, rural, low-income adults.

## Methods

### Study site, population, and survey

The average resident in the four Indiana counties chosen for study (Crawford, Greene, Lawrence, Orange) before the pandemic was older, had less income, and was equally or less food secure than the average Indiana resident and the national average [22, 23]. The studied counties are considered “mostly rural” or “rural” [24], and the counties lack racial and ethnic diversity, each being over 96% white [23]. Given the population of interest, we opted for a paper survey to be mailed to households in the four counties rather than a web-based survey, which would be less likely to reach many residents due to lower uptake of internet and computer technology among this demographic [25] and a digital divide, especially in Crawford county where, as of 2018, over one-third of households had no internet access and broadband access was almost completely unavailable [26].

We purchased an address-based sample (ABS) list of 5,000 residential households from Marketing Systems Group to which we mailed the surveys. The ABS covers nearly all households in the United States and is built using the USPS Computerized Delivery Sequence File. While the ABS frame is probability based, the sample for this study was non-probability, targeted to households with members aged 60 or older and at or below 185% of the poverty threshold.

The survey instrument asked respondents to report on their experiences both prior to the pandemic and since the pandemic began. In addition to the HFSSM, HRQOL-4, and UCLA Revised Loneliness Scale, the survey covered a range of questions having to do with food security, food provisioning strategies, and health and well-being before and since the COVID-19 pandemic began. The survey was developed after initial focus groups with older adults in the four counties, and then the survey was piloted with a small convenience sample of older adults, with a member of the research team having an in-depth conversation with these piloting respondents to understand any confusing question or other factors in the survey that might be improved. A pre-survey notification in the form of a postcard was mailed on May 6, 2020, and the paper survey on May 8, 2020. A reminder postcard was mailed one week later. Respondents would have received the survey shortly after the statewide shelter-in-place order had been lifted and reopening of the state had begun for most counties on May 4, 2020 (Indiana Executive Orders 20–08, 20–26). This study was approved by Indiana University’s Institutional Review Board (#2008204182). Respondents were not required to provide informed consent. All respondents received a study information sheet (mailed with the paper survey) that informed them of the purpose of the study, provided a statement that their participation is voluntary, and contact information for the principal investigator. The research team accepted survey responses until August 20, 2020. We incentivized all potential respondents with a $5 grocery store gift card included in the paper survey packet. The participation rate for the survey was 29.6% (1481 people).

### Variables and statistical methods

The HFSSM asks how often respondents found themselves in certain food-related situations in the last twelve months, the HRQOL asks about physical and mental health in “a typical month,” and how many days in that period a person feels their physical and mental health were not good (i.e., “unhealthy days”). It is a subjective measure of one’s health status. The Loneliness Scale asks generally, without any specified time frame, how often a person feels lonely and isolated. Survey questions were further qualified in terms of both before COVID-19 and since COVID-19. Wording for all survey questions analyzed in this paper can be found in S1 Appendix. Scores for HFSSM are assigned on a scale of 0–6 as follows: 0–1—High or marginal food security; 2–4—Low food security; 5–6—Very low food security. From the HRQOL-4, we calculated the number of unhealthy days for each respondent in terms of their physical and/or mental health. The loneliness scale assigns scores on a scale of 3 (not lonely) to 9 (very lonely).

Before statistical analysis, we excluded any respondents who were under the age of 60, which left 1401 respondents. We used the R Base package and car package in analysis [27, 28]. We used a binomial test to see whether there were statistically significant changes for the population since COVID-19 began for the three measures of interest: food security, unhealthy days, and loneliness. Then, we created three separate logistic regression models to understand which variables were associated with scores becoming worse for each metric. To create dependent variables denoting reported change, we first calculated the difference in each person’s score for food security, unhealthy days, and loneliness prior to and after the pandemic began, and then binarized those, where 0 = no change or improvement in score and 1 = score worsened. The number of people whose scores improved was very small (1.8%, 3.1%, and 1.1% of the sample for food security, healthy days, and loneliness respectively) so we combined them with people for whom there was no change in score.

We identified predictors of change in perceived well-being as follow. We use four structural variables that cover limitations to transportation, operating hours for grocery stores, the closing of food pantries, and to what extent stores ran out of food that respondents typically purchase. In addition, we include responses to two questions that relate to personal perceptions of risk and fear: to what degree COVID-19 made a person feel afraid to go to the grocery store or pantry, and to what degree a person has stockpiled supplies. Respondents were to interpret “supplies” as whatever it meant to them.

These variables were used purposively across the three models. We consider that for food security, all these COVID-19-related variables could impact one’s ability to procure food. For unhealthy days, we include the transportation variable, the fear variable, and the stockpiling variable. Lack of transportation would decrease access to spaces, activities, and resources that might affect perceived physical and mental health. We consider the fear and stockpiling variables to be indicators of stress affecting mental health. For the loneliness model, we include the transportation and fear variables. With in-person interactions as restricted as they were at the time of the survey, any kind of interaction with others, even as fleeting as in a grocery store, could have helped people feel less lonely, and thus the ability to get to places like this might have mattered. Also in the loneliness model, we include a variable that measures the change in how often people ate with others before and since the pandemic began. We asked, “In a typical week before the COVID-19 outbreak, how often did you eat with others?” (Always, Usually, About half the time, Seldom, Never). We coded the difference before and since COVID-19 in terms of three categories: eating less frequently with others, no change in frequency of eating with others, and eating more frequently with others since the pandemic.

In all models, we also included demographic variables for respondent age, gender, education, marital status, impact of the pandemic on income, and ethnic and/or racial background. No respondent identified as anything other than male or female. We asked respondents to select any of ten (in addition to a write-in option and Prefer not to respond) ethnic and/or racial backgrounds that applied to them: African American or Black, White Caucasian non-Hispanic, Hispanic or Latinx, Pacific Islander or Native Hawaiian, American Indian or Native Alaskan, East Asian, Southeast Asian, Indian, Middle Eastern, African. For this analysis, because there were so few respondents who selected anything other than White Caucasian non-Hispanic, we coded respondents white if they only selected “White Caucasian—Non-Hispanic” and non-white if they selected any other options/write-in that indicated another group.

All logistic regression models were tested for multicollinearity using the variance inflation factor, all values of which were less than 1.5 in all models. We also tested the models for overdispersion by looking at the ratio of the residual deviance to residual degrees of freedom, and all values were under 1.3 (0.26, 1.16, and 1.29 respectively for food security, unhealthy days, and loneliness models). Based on focus group results from an earlier stage of this study that indicated transportation being a barrier for food security and social interaction for certain types of adults in this sample [29], we included an interaction term for age and the transportation variable in each model. This term was not significant in any model so we removed it from the final models.

## Results

The binomial tests indicate that the shifts in scores were all statistically significant (Table 1). We note as well that the prevalence of food insecurity in this sample was low, both before and since the pandemic began. The proportion of respondents reporting very low food security changed from 1.3% to 1.6%, and those reporting low food security changed from 4.3% to 4.5% (thereby leaving 94% of respondents and 93.9% of respondents with high or marginal food security before and since COVID-19, respectively). The mean scores for food security, unhealthy days, and loneliness all increased, that is, food security became worse and people reported more unhealthy days and more loneliness, since the pandemic began.

**Table 1:** Scores, change in the measures of interest, and *p*-values for binomial tests of significance in change in scores before and since the pandemic began.

	Mean (SD) score before COVID-19	Mean (SD) score since COVID-19	Percent respondents whose score worsened	*p* (binomial test)
Food security (n = 1395)	0.25 (.8)	0.28 (1)	4%	< .001
Unhealthy days (n = 1327)	5.9 days (9.5)	7.8 days (10.5)	29%	< .001
Loneliness (n = 1311)	3.8 (1.4)	5.0 (2)	51%	< .001

Scores for food security are assigned on a scale of 0–6: 0–1—High or marginal food security; 2–4—Low food security; 5–6—Very low food security. The score for unhealthy days is the number of unhealthy days in the last 30 days in terms of physical and/or mental health. The loneliness scale assigns scores on a scale of 3 (not lonely) to 9 (very lonely). The scores for unhealthy days and loneliness are not qualitatively interpreted any further (e.g., good or poor health). For all measures, both pre-pandemic and since the pandemic began, respondents exhibited the full range of scores.

Table 2 provides descriptive statistics for the demographic variables and the variable for eating with others. The average respondent was just over 71 years old. Most respondents were female and overwhelmingly identified as white. Over 40% of the sample had no more than a high school education, and nearly two-thirds were married or living with a partner. During the pandemic, nearly half of respondents reported eating less often with others, with the vast majority of the remainder reporting no change in frequency.

**Table 2:** Descriptive statistics for demographic variables included in statistical models.

Variable	Activity and respondents reporting (%)		Mean (SD)	N
Age			71.4 (7.6)	1401
Change in eating with others	Eating less often with others	49.1%		1355
	No change in frequency	48.9%		
	Eating more often with others	2.1%		
Gender	Female	71.4%		1395
Ethnic and/or racial background	Non-white	3.5%		1234
Education	Less than high school	2.2%		1277
	Some high school	4.2%		
	High school diploma/GED	38.6%		
	Trade certification	6.7%		
	Some college	22.9%		
	College degree	14.4%		
	Post college degree	9.7%		
Marital status	Married or living with partner	64.7%		1344
	Widowed	21.0%		
	Divorced or separated	11.4%		
	Never married	3.0%		

Note: n changes for each variable due to missing data.

**Fig 1:**
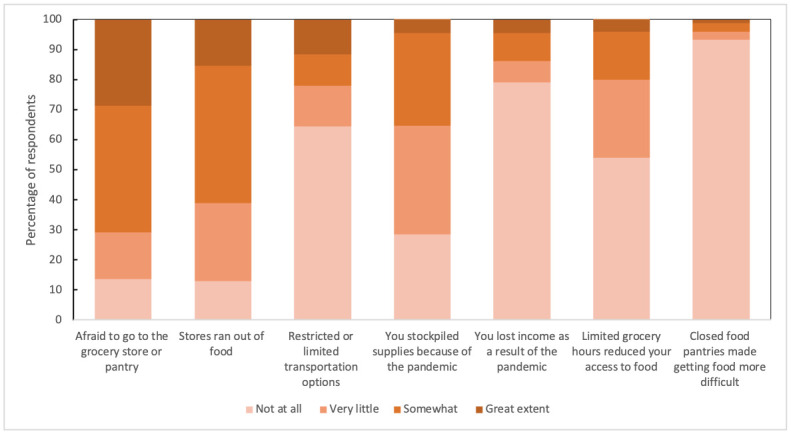
Descriptive statistics for pandemic-related variables used in the models.

Fig 1 provides descriptive statistics for the COVID-19-related variables. Very few respondents reported being impacted by food pantries closing, and over half of respondents were not impacted by the pandemic in terms of transportation, limited grocery hours, or loss of income. However, over a quarter were afraid to go to the store or food pantry, and stores running out of food affected over 85% of the sample. Nearly three-quarters of respondents stockpiled food to some degree.

Being afraid to go to the grocery store or pantry was significant across all three models, and in all models, this was associated with higher odds of worse food security, more unhealthy days, and more loneliness. Two additional structural variables relating to access to food, closed pantries and limited grocery store hours, were significantly associated with higher odds of reduced food security. A combination of demographic variables and COVID-19-related variables was significant for the unhealthy days model. Being more educated, younger, and female all predicted greater odds of an increase in perceived unhealthy days, as did having one’s income impacted by the pandemic and more stockpiling behavior. For loneliness, being younger increased the odds of feeling more loneliness. Limited transportation also affected the odds of greater loneliness during the pandemic. Finally, those who reported no change in their frequency of eating with others were less likely to feel lonelier, compared to respondents who ate with others less often during the pandemic. In Table 3 we present adjusted odds ratios (AOR) with 95% confidence intervals for the three logistic regression models and indicate which variables were statistically significant in the models.

**Table 3:** Adjusted odds of worse food security, more unhealthy days, and more loneliness.

	Food security (n = 1032) AOR (95% CI)	Unhealthy days (n = 1140) AOR (95% CI)	Loneliness (n = 1083) AOR (95% CI)
Education	1.00 (0.80, 1.25)	1.12 (1.02, 1.22)*	1.09 (1.00, 1.18)
Age	1.03 (0.98, 1.08)	0.97 (0.95, 0.99)*	0.98 (0.96, 1.00)*
Gender			
Male	1 (Ref)	1 (Ref)	1 (Ref)
Female	0.90 (0.40, 2.00)	1.94, (1.40, 2.68)***	1.22 (0.91, 1.62)
Marital status			
Married/living with partner	1 (Ref)	1 (Ref)	1 (Ref)
Widowed	1.33 (0.56, 3.15)	1.26 (0.87, 1.83)	0.99 (0.70, 1.40)
Divorced or separated	0.69 (0.20, 2.39)	1.00 (0.63, 1.57)	0.96 (0.63, 1.45)
Never married	2.20 (0.44, 11.08)	1.29 (0.59, 2.84)	0.54 (0.25, 1.16)
Ethnic/racial background			
White	1 (Ref)	1 (Ref)	1 (Ref)
Non-white	0.33 (0.03, 3.20)	0.75 (0.31, 1.85)	1.41 (0.65, 3.08)
Afraid to go to store	2.44 (1.38, 4.30)**	1.29 (1.11, 1.52)**	1.30 (1.13, 1.49)***
Limited transport	0.87 (0.64, 1.20)	1.13 (0.99, 1.30)	1.14 (1.00, 1.31)*
Impact on income	1.29 (0.93, 1.79)	1.18 (1.02, 1.38)*	1.07 (0.92, 1.25)
Stockpiling	1.44 (0.97, 2.13)	1.31 (1.11, 1.54)**	
Limited grocery hours	2.21, (1.47, 3.33)***		
Stores run out of food	1.51 (0.91, 2.48)		
Closed pantries	1.66 (1.11, 2.49)*		
Change in eating with others			
Eating less often with others			1 (Ref)
No change			0.37 (0.28, 0.48)***
Eating more often with others			0.68 (0.29, 1.62)

Note:

* *p* < .05,

** *p* < .01,

*** *p* < .001

n varies for each model due to listwise deletion of cases.

## Discussion

The COVID-19 pandemic negatively impacted multiple aspects of well-being in this sample of older, lower income, rural dwelling adults, and a mix of structural, risk, and demographic factors were related to worse food security, more unhealthy days, and more loneliness. The three measures we considered all became statistically significantly worse for respondents. Given the attention that food insecurity has rightfully received during the pandemic, combined with the fact that people living in rural areas are more likely to experience food insecurity in general both during and before the pandemic [30], we might have expected more people to report worse food security than did. However, it may be that a significant number of older adults were insulated from food insecurity because of aspects of the social safety net, such as income from social security. The increase in unhealthy days and loneliness are consistent with other studies documenting the impacts of COVID-19 [31].

Structural factors, particularly limited grocery store hours and the closure of pantries, were key for food security. Large grocery chains that were open 24 hours prior to the pandemic abruptly cut hours, and some local shops that carried food products closed permanently. Food pantry accessibility in these counties is highly variable at the best of times [32], and access to fresh vegetables, fruit, and meat is problematic for lower income households in parts of these counties [33]. Any significant changes in operations for pantries or groceries in the area could impact food security.

The period during which we collected these data, that is, immediately after shelter-in-place orders were lifted in spring of 2020, was a time when people would have had to navigate a new landscape of risk and uncertainty related to food procurement and social interaction. In addition to affecting food procurement, fear of going to the store or pantry may be a proxy for general feelings of fear of going out, and therefore would impact our other two measures as well. This corresponds with findings by Fitzpatrick and colleagues [34], who found that people reporting more fear associated with COVID-19 also reported more symptoms of poor mental health. Fear contributes to more unhealthy days in this sample, and fear could contribute to increased feelings of isolation and being left out, both components of the loneliness scale. Stockpiling of supplies is an expression of risk perception, and stockpiling behavior has been found to be more frequent among those more worried about the pandemic [35].

Those who are more educated may have been more likely to see an increase in unhealthy days because lockdowns and social distancing restricted opportunities for things like exercise and preventive care, which those with more education are more likely to do [36]. A tendency for those with more education to be in better health overall, that is, to have fewer unhealthy days to begin with, could also have contributed to greater odds of reporting more unhealthy days. That women were also more likely to report an increase in this measure is in line with studies finding discrepancies in impact of the pandemic by gender [37]. While a loss of income due to the pandemic was not significantly associated with increased odds of worse food security, it was associated with an increase in the odds of reporting more unhealthy days. We consider that in this context, the stress of that loss impacted the mental health aspect of the unhealthy days measure. What might seem to be counterintuitive negative associations between age and increased loneliness and age and more unhealthy days have in fact been documented in other populations [38, 39]. Such an association between age and the mental health aspect of unhealthy days also aligns with what some term a paradox of aging: while the size of one’s social networks tends to shrink with age, incidence of mental health disorders tends to be lower and feelings of well-being increase [40].

Although opportunities to see others would still have been reduced at the time of the survey, fear, coupled with a lack of transportation, would have restricted the potential for direct interpersonal interaction, thereby contributing to loneliness. Finally, those who maintained the frequency with which they ate with others were less likely to report increased loneliness, underscoring the multiple roles food plays in our lives. Humans tend to eat together [41], and suddenly being unable to do this as often could certainly contribute to feelings of loneliness. The onset of the pandemic provided a natural experiment that reduced some older adults’ opportunities to take meals with others, a pattern which in this sample was associated with increased loneliness among the 49% who ate with others less after the start of the pandemic than they did prior. This finding echoes prior work [29] and suggests some of the value to mental health that congregate meals, such as those stipulated under the Older Americans Act, may provide to support the resilience of older adults.

## Limitations

The study is cross-sectional in nature, asking respondents to recall their typical experiences and feelings before the pandemic and since the pandemic began within a single instrument. As with most survey research, recall error can be a source of bias, and an individual’s assessment of their past state can be influenced by their current state. The longer the period of recall, the more likely a respondent will misreport. In this study, the window for recall would have been, at shortest, approximately six to eight weeks. In mid-March, the WHO declared COVID-19 to be a global epidemic and the U.S. government declared a national emergency, and in late March, the governor of Indiana declared a stay-at-home order (Indiana Executive Order 20–08). Recall that respondents received the survey in early to mid-May. We have no way to assess to what degree recall bias might have influenced respondents’ perceptions. In addition, ideally, we would have used the longer forms of the HFSSM and Loneliness Scale, but in the interest of lessening respondent burden, we chose to use the shorter forms.

The sample of addresses that we purchased is not guaranteed to be 100% accurate in terms of the target variables (age 60+, at or below 185% of the poverty threshold). While we excluded any respondent who was under the age of 60 from this analysis, we did not ask for income information at a scale that allowed us to exclude households who did not meet the poverty threshold requirement (i.e., response options were income ranges). Therefore, the analysis may have included some households above our target income threshold and we cannot estimate to what extent this may have happened.

We recognize that during the pandemic information changed week to week, even day to day, resulting in many respondents’ situations being in flux. While we asked respondents to indicate the date they filled out the survey, many did not do this, and so we were unable to meaningfully analyze change over time at a finer scale than we have done here without significantly reducing the sample size, thus we are largely unable to capture this flux.

Finally, due to the self-administration method of the survey, we may not have captured respondents who are illiterate or have low literacy.

## Conclusion

Our analysis focused on a sample of people who not only would be expected to be most vulnerable to COVID-19 and its health impacts, but who have specific health and dietary needs outside of a pandemic. In order for rural residents to be able to age in place well, for the U.S. to repopulate and revitalize rural areas, and to close gaps in morbidity and mortality in rural places, then food, health care, and public health systems must be reshaped. The results presented here highlight certain impacts the pandemic has had on older, rural, lower income adults, and the different factors that are associated with diminished well-being in the first months of the pandemic. Understanding these associations and what resources will be needed for people to recover from this ongoing pandemic, and to better endure future ones, can be a place to begin this reshaping. Rural residents will need resources to recover both physically and mentally from the pandemic, and solutions should be designed that will mitigate any far-reaching negative impacts of the pandemic and that will help establish (or re-establish) infrastructure to support rural residents in the future. We propose a number of ways to start: developing a spatiotemporal lens on food retail [32] and emergency food outlets [31] for rural residents; redoubling public and private support of congregate meal opportunities for older adults [29]; improving rural transportation; and a renewed and coordinated focus on increasing healthcare, educational and cultural services in rural America.

## Supporting information

S1 Appendix Survey questions used in analysis. (PDF)
