# Supplementary material for: Food security and well-being among older, rural Americans before and during the COVID-19 pandemic
Source: PLoS One. 2022 Sep 2;17(9):e0274020. doi: 10.1371/journal.pone.0274020 (PMC9439215; doi:10.1371/journal.pone.0274020)
Supplement: S1 Appendix — (PDF) [file pone.0274020.s001.pdf]

Please read the statements below and answer whether each was often, sometimes, or never true for you or your household in a typical month before the COVID-19 outbreak and then since the COVID-19 outbreak.

“The food that (I/we) bought just didn’t last, and (I/we) didn’t have enough money to get more.” Was that often, sometimes, or never true for (you/your household) in a typical month before the COVID-19 outbreak

- Often true
- Sometimes true
- Never true
- Don't know or refuse to answer

“The food that (I/we) buy just doesn’t last, and (I/we) don’t have enough money to get more.” Is that often, sometimes, or never true for (you/your household) *since the COVID-19 outbreak*

- Often true
- Sometimes true
- Never true
- Don't know or refuse to answer

“(I/we) couldn’t afford to eat balanced meals.” Was that often, sometimes, or never true for (you/your household) *in a typical month before the COVID-19 outbreak*? You can define balanced meal in whatever way you think is best.

- Often true
- Sometimes true
- Never true
- Don't know or refuse to answer

"(I/we) can't afford to eat balanced meals." Is that often, sometimes, or never true for (you/your household) *since the COVID-19 outbreak*? You can define balanced meal in whatever way you think is best.

- Often true
- Sometimes true
- Never true
- Don't know or refuse to answer

In a typical month before the COVID-19 outbreak, did you or other adults in your household ever cut the size of your meals or skip meals because there wasn't enough money for food?

- Yes
- No
- Don't know or refuse to answer

Since the COVID-19 outbreak do you or other adults in your household ever cut the size of your meals or skip meals because there isn't enough money for food?

- Yes
- No
- Don't know or refuse to answer

In a typical month before the COVID-19 outbreak, how often did you or others in your household cut the size of meals or skip meals because there wasn't enough money for food?

- Almost every week
- Some weeks but not every week
- Only once or twice a month
- Don't know or refuse to answer

Since the COVID-19 outbreak, how often do you or others in your household cut the size of your meals or skip meals because there isn't enough money for food?

- ◯ Almost every week
- ◯ Some weeks but not every week
- ◯ Only once or twice a month
- ◯ Don't know or refuse to answer

In a typical month before the COVID-19 outbreak, did you or others in your household ever eat less than you felt you should because there wasn't enough money for food?

- ◯ Yes
- ◯ No
- ◯ Don't know or refuse to answer

Since the COVID-19 outbreak, do you or others in your household ever eat less than you feel you should because there isn't enough money for food?

- ◯ Yes
- ◯ No
- ◯ Don't know or refuse to answer

In a typical month before the COVID-19 outbreak, were you or others in your household ever hungry but didn't eat because there wasn't enough money for food?

- ◯ Yes
- ◯ No
- ◯ Don't know or refuse to answer

Since the COVID-19 outbreak, are you or others in your household ever hungry but don't eat because there isn't enough money for food?

- Yes
- No
- Don't know or refuse to answer

Thinking about your physical health, which includes physical illness and injury, for how many days during a typical month (before COVID-19) was your physical health not good? Number of days:

Thinking about your physical health, which includes physical illness and injury, for how many days during the past month (since COVID-19) was your physical health not good? Number of days:

Now thinking about your mental health, which includes stress, depression, and problems with emotions, for how many days during a typical month (before COVID-19) was your mental health not good?

Now thinking about your mental health, which includes stress, depression, and problems with emotions, for how many days during the past month (since COVID-19) was your mental health not good?

Before COVID-19, how often did you feel that you lacked companionship?

- Hardly ever
- Some of the time
- Often

Since COVID-19, how often do you feel that you lack companionship?

- Hardly ever
- Some of the time
- Often

Before COVID-19, how often did you feel left out?

- ◯ Hardly ever
- ◯ Some of the time
- ◯ Often

Since COVID-19, how often do you feel left out?

- ◯ Hardly ever
- ◯ Some of the time
- ◯ Often

Before COVID-19, how often did you feel isolated from others?

- ◯ Hardly ever
- ◯ Some of the time
- ◯ Often

Since COVID-19, how often do you feel isolated from others?

- ◯ Hardly ever
- ◯ Some of the time
- ◯ Often

The next few questions relate to the recent COVID-19 *outbreak*. For each question, please check the box that applies.

	To a great extent	Somewhat	Very little	Not at all
How afraid are you to go to the grocery store or pantry because of the COVID-19 outbreak?	○	○	○	○
How much has the COVID-19 outbreak restricted or limited your transportation options?	○	○	○	○
How much have limited grocery hours reduced your access to the foods you typically purchase?	○	○	○	○
To what extent have stores or pantries run out of the foods you typically get?	○	○	○	○
Has the closing of food pantries made getting food more difficult?	○	○	○	○
Have you lost income as a result of the COVID-19 outbreak?	○	○	○	○

To what extent have you stockpiled supplies because of the COVID-19 outbreak?	○	○	○	○

In a typical week before the COVID-19 outbreak, how often did you eat with others?

- ○ Always
- ○ Usually
- ○ About half the time
- ○ Seldom
- ○ Never

Since the COVID-19 outbreak, how often do you eat with others?

- ○ Always
- ○ Usually
- ○ About half the time
- ○ Seldom
- ○ Never
